# High-Sensitivity Air-Coupled Ultrasonic Transducer Design Based on PMN-PT Bending Vibration Modes

**DOI:** 10.3390/s25226912

**Published:** 2025-11-12

**Authors:** Junwen Deng, Xingyu Chen, Yuliang Zhu, Xiaobo Wang, Tao Han, Chengtao Luo

**Affiliations:** School of Automation and Intelligent Sensing, Shanghai Jiao Tong University, Shanghai 200240, China; cross_sada@sjtu.edu.cn (J.D.); chenxingyu9@sjtu.edu.cn (X.C.); zhuyl2@sjtu.edu.cn (Y.Z.); 022035910015@sjtu.edu.cn (X.W.);

**Keywords:** gas leak detection, ultrasonic transducer, air-coupled, bending vibration modes, high sensitivity

## Abstract

Gas pipelines are a critical means of transportation in industrial production. To detect gas pipeline leaks, ultrasonic transducers with specific center frequencies and high sensitivity are required. This paper proposes a novel air-coupled ultrasonic transducer design based on a horn-type matching layer and a bending-mode type of piezoelectric material, specifically tailored for gas leak detection scenarios. The transducer design is optimized by the finite element method, focusing on the basic components of the piezoelectric bimorph, the horn and the supporting tube. First, the influence of various dimensional parameters of the piezoelectric bimorph on the bending vibration mode was analyzed. Then, the effects of the other two components, the horn and the supporting tube, on the piezoelectric bimorph vibration mode were investigated. A parametric scan on the dimensions of these components was conducted to optimize the transducer’s output. Finally, ultrasonic transducers using PMN-PT and PZT were fabricated and tested. The results show that the sensitivity of those transducers surpasses that of similar commercial transducers, especially the PMN-PT one with a center frequency of 40 kHz and a peak receiving sensitivity of −51.1 dB. This transducer, benefiting from the high-performance piezoelectric material and the bending vibration mode, proves to be a promising candidate for high-precision leak detection in gas pipelines.

## 1. Introduction

Pipeline is a primary mode for gas transportation within the modern industrial and energy sectors [1]. Since the working conditions of gas pipelines are commonly subjected to high pressure, corrosion, and wear, the corresponding material aging, fatigue, and inherent defects will inevitably lead to pipeline fractures [2,3,4]. Thus, the real-time leak detection for gas pipeline fractures is essential for ensuring the manufacturing safety and efficiency [5,6].

Generally, the leak detection should exhibit high accuracy, adaptability, and stability. Currently, the common methods for gas pipeline leak detection include ultrasonic detection [7], acoustic emission detection [8], negative pressure wave measurement [9], distributed fiber optic sensing systems [10], and infrared imaging detection [11]. Among these methods, the ultrasonic detection utilizes an air-coupled ultrasonic transducer to capture airborne ultrasound [12], which arises when pressurized gas inside the pipeline escapes through leaks. Apart from its non-contact feature, ultrasonic detection stands out through its advantages of high sensitivity and resistance to electromagnetic interference, making it preferred in many complex and harsh working conditions, such as detecting gas pipeline leaks in factories or on ships.

The air-coupled ultrasonic transducer is the core component of the ultrasonic leak detection system, and it requires high sensitivity and appropriate operating frequencies to capture the ultrasonic leak signals effectively. At present, ultrasonic transducers can be broadly categorized into capacitive and piezoelectric ones. The capacitive transducers offer advantages such as flexible geometric design and on-chip electronic integration. However, their sensitivity is generally lower than that of the piezoelectric transducers [13]. In addition, the piezoelectric transducers provide broader bandwidth and better signal-to-noise ratio (SNR) [14].

The development of the piezoelectric transducer is mainly focused on its two functional components: piezoelectric materials and matching layers. First of all, the piezoelectric material converts the input acoustic signal into an output electrical signal via the piezoelectric effect. Using high-performance piezoelectric materials can significantly enhance the transducer sensitivity. For example, Song et al. [15] developed a highly sensitive ultrasonic transducer by adopting the high-performance PMN-PT single crystal as the piezoelectric material, outperforming traditional ones using PZT ceramics. Secondly, the matching layer collects the airborne ultrasonic signals and transmits them to the piezoelectric material. After the piezoelectric materials are determined, the acoustic impedance of the matching layer is designed to achieve high transmission efficiency, and it should be set between that of the air and the piezoelectric materials. However, there is a significant acoustic impedance mismatch between the air (400 Rayl) and the piezoelectric materials (28.8 MRayl for PZT, and 28.0 MRayl for PMN-PT), which makes the most collected ultrasonic signals reflected and lost before reaching the piezoelectric material. Thus, enhancing the transducer’s sensitivity solely by modifying the matching layer is particularly challenging [16]. One of the solutions is employing the novel piezoelectric materials with lower acoustic impedance, or utilizing multiple matching layers with a gradient acoustic impedance. For instance, Li et al. [17] combined both optimization methods mentioned above by using 1–3 piezoelectric composite materials (14Mrayl) and double matching layers in their transducer design. This makes their transducer exhibit an insertion loss 3.8 dB lower than the traditional ones, which can mainly be attributed to an optimized acoustic transmission efficiency. Nevertheless, the fabrication process is complicated and costly for those low-impedance matching layers, and the bonding materials between the multi-layer matching layers can also diminish the acoustic transmission efficiency [18]. Therefore, the horn was proposed as another matching structure for air-coupled ultrasonic transducers, which is intended to capture and concentrate ultrasonic energy in the air. For example, Meisel et al. [19] designed and optimized a horn using a finite element method (FEM) model, which significantly improved the directivity and sensitivity of ultrasonic transducers. Darras et al. [20] tested the ultrasound transmission loss and directivity of ultrasonic transducers with and without horns. Results show that the horns can substantially improve the transmission efficiency of the ultrasonic signal, thus enhancing the performance of the transducer. Nonetheless, these studies focus on improving the acoustic capture ability of the horn, but rarely discuss its influence on the working mode of the piezoelectric vibrators and the impact of its dimensional parameters on transducer output.

Besides, the piezoelectric vibrators of the air-coupled transducers mentioned above are mostly operated under the thickness-mode or length-mode. However, the vibrators operating under these modes are oversized for most transducers, since their resonance frequencies are around 40 kHz, which is required most for gas pipeline leak detection [21]. In this case, the piezoelectric vibrators are usually designed in the bending vibration mode for the low-frequency requirement. For example, Zhang et al. [22] introduced a transducer design leveraging the bending vibration mode of piezoelectric bimorphs. By optimizing the matching layer structure, they developed a low-frequency ultrasonic transducer capable of detecting failure signals of rotating machinery. While such studies have applied the bending vibration mode to the contact transducers, few have explored it in air-coupled transducers. Methods for designing the center frequency of air-coupled transducers using appropriate piezoelectric elements in bending vibration modes remain to be discussed.

In summary, this paper proposes a novel air-coupled ultrasonic transducer design based on a horn-type matching layer and a bending vibration mode type of piezoelectric materials, specifically tailored for gas leak detection scenarios. Based on the FEM and parameter optimization, the transducer was then fabricated and tested. This work validates the feasibility of designing air-coupled ultrasonic transducers for pipeline leak detection by utilizing a bending-mode piezoelectric vibrator combined with a horn, thereby laying a solid foundation for the development of a real-time pipeline leak detection system.

## 2. Materials and Methods

### 2.1. Simulation Environment

To investigate the influence of different components of the air-coupled ultrasonic transducer on its output characteristics, simulations of the key components were conducted using the COMSOL 6.1 finite element analysis software. The air-coupled ultrasonic transducer consists of a piezoelectric bimorph, a horn, a supporting tube, and a shell. The simulation work in this study focuses on optimizing the transducer output by changing dimensions and working load of the piezoelectric bimorph. The parameters used are shown in Table 1.

To accelerate the simulation process, the modeling of the shell, horn, and supporting tube was excluded in the FEM analysis, and the mechanical constraints and boundary conditions were applied to the piezoelectric bimorph directly.

### 2.2. Fabrication of Ultrasonic Transducers

The properties of the piezoelectric material, as the functional component, are of critical importance when fabricating high-performance ultrasonic transducers. Compared to the widely used PZT-5H ceramic, PMN-xPT$\left((1-x)Pb({Mg}_{1/3}{Nb}_{2/3}O_{3}-x{PbTiO}_{3},x=0.29\right)$ single-crystal material exhibits substantially higher piezoelectric coefficients and electromechanical coupling efficiency, which can significantly enhance the sensitivity of the transducer. In this study, the PMN-xPT (x = 0.29) single-crystals (produced by Innovia Materials (Shanghai) Co., Ltd., Shanghai, China) were polarized along the [001] direction in thickness mode and cut into circular thin plates with a diameter of 10 mm and a thickness of 0.4 mm.

The fabrication process of the ultrasonic transducer is as follows: First, two piezoelectric plates (10 mm × 0.4 mm) are bonded together with conductive adhesive with their polarization directions opposite. The assembly is then heated in an oven at 60 °C for 6 h to ensure the adhesive layer is fully cured and the piezoelectric bimorph is completely coupled. Epoxy resin is applied to both ends of the supporting tube, with one end bonded to the casing and the other bonded to the bimorph. It is ensured that the center of the bimorph aligns with the axis of the supporting tube. Finally, epoxy resin is applied to the base of the horn, which is then bonded to the center of the bimorph. It is important to note that, to prevent the PMN-PT material from depolarizing at high temperatures and losing its piezoelectric properties, the transducer avoids the use of soldered wires. Instead, conductive adhesive is used to connect the wiring. Two transducers were thus fabricated using PMN-PT and PZT materials, respectively, and their output performance was tested and compared.

### 2.3. Transducer Testing System

In this work, the sensitivity of the transducer is defined as the ratio of the output voltage to the received sound pressure, as is shown in Equation (1):

(1) $$S_{p}\left(\omega \right)=20{log}_{10}\frac{V\left(\omega \right)}{P\left(\omega \right)}$$

In which $V\left(\omega \right)$ represents the output voltage of the transducer, and $P\left(\omega \right)$ represents the sound pressure received by the transducer. This sensitivity can be measured through the calibration test of the transducer.

**Figure 1:**
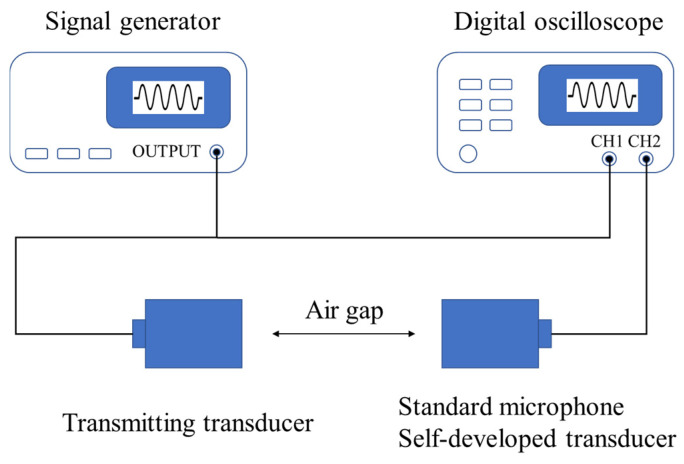
Experimental set-up scheme for transducer characterization.

The receiving sensitivity of the air-coupled ultrasonic transducer was tested using the calibration method, and the test system is shown in Figure 1. The ultrasonic transducer (PAC 40K-54N-T(R), Physical Acoustics Technology, Beijing, China) and the standard microphone were fixed along the same axis. A signal generator (Tektronix AFG1022, Tektronix, Beaverton, OR, USA) was used to generate signals of different frequencies to excite the ultrasonic transducer to emit ultrasound, while an oscilloscope (Tektronix DPO3045, Tektronix, Beaverton, OR, USA) recorded the output voltage of the standard microphone at different frequencies. The standard microphone was then replaced with the self-developed ultrasonic transducer, and the above measurement steps were repeated to obtain the output voltage of the self-developed ultrasonic transducer at different frequencies. The sensitivity of the self-developed ultrasonic transducer was calculated based on the known sensitivity of the standard microphone using the conversion formula shown in Equation (2).

(2) $$S_{u}=S_{m}-20*{log}_{10}\left(\frac{V_{m}\left(\omega \right)}{V_{u}\left(\omega \right)}\right)$$

In which, $S_{u}$ represents the sensitivity of the self-developed transducer, $S_{m}$ represents the sensitivity of the standard microphone, $V_{m}\left(\omega \right)$ is the output voltage of the standard microphone, and $V_{u}\left(\omega \right)$ is the output voltage of the self-developed transducer. The impedance characteristics of the ultrasonic transducer were evaluated using the Agilent 4294A impedance analyzer (Agilent, Santa Clara, CA, USA).

## 3. Results

### 3.1. Basic Components and Functions of Ultrasonic Transducers

Ultrasonic signals generated by gas leaks are often weak and attenuate rapidly as they propagate through the air. Existing studies have shown that for gas leak signals, the sound intensity around 40 kHz is significantly higher than environmental noise [23]. To improve signal reception efficiency and suppress noise interference, the center frequency of the transducer should be set as close to 40 kHz as possible while ensuring sensitivity.

As the functional component of the ultrasonic transducer, the structure of the piezoelectric vibrator fundamentally determines the transducer’s center frequency and receiving sensitivity. In this study, we applied a bimorph working under the bending vibration modes as the piezoelectric vibrator. By bonding two circular piezoelectric thin plates with opposite polarization directions, a low-frequency bending vibration mode can be excited. As both piezoelectric thin plates generate an electrical signal while working, this bimorph provides a higher electrical output compared to those using a single plate. The transducer’s center frequency is controlled by adjusting the geometric dimensions of the bimorph, which also optimizes the characteristics of the bending vibration mode to achieve better electrical output. Design and optimization details are discussed in the following section.

To improve the receiving sensitivity of the ultrasonic transducer, two components, a horn and a supporting tube, were designed to further excite the bending vibration mode of the piezoelectric bimorph. The disk-shaped horn collects ultrasonic waves from the air and converts the sound pressure into a force that acts on the center of the piezoelectric bimorph. The supporting tube, designed in a tubular shape, provides mechanical support for the piezoelectric vibrator. These two components add a pair of oppositely directed forces on the center and the edge of the piezoelectric bimorph, respectively, during operation, thereby better exciting the bending vibration mode. Adjusting the dimensions of the horn and supporting tube changes the excitation level of the bending vibration mode, and optimization of these components is further discussed in subsequent sections.

**Figure 2:**
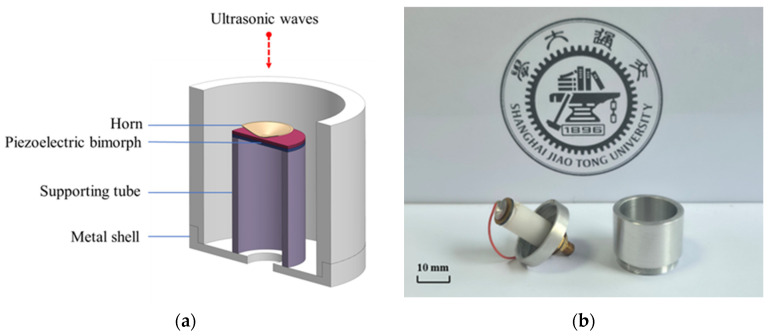
(**a**) Structural design of ultrasonic transducer; (**b**) photograph of the ultrasonic transducer.

In summary, the overall structure of the transducer, as shown in Figure 2a, includes the piezoelectric bimorph, horn, supporting tube, and metal shell. Figure 2b shows the photograph of the ultrasonic transducer. This design effectively detects ultrasonic leakage signals in the air and ensures reliable performance in complex industrial environments.

### 3.2. Design of the Center Frequency for Ultrasonic Transducers

For the receiving sensor, working at the anti-resonance frequency is advantageous for enhancing sensitivity. In traditional ultrasonic transducer designs, to maximize the output of the vibrator, the piezoelectric vibrator is typically designed to operate in the length mode (33 mode), where the piezoelectric coefficient is at its maximum. In this mode, the length of the piezoelectric vibrator becomes the primary dimension controlling its resonance and anti-resonance frequencies. As the primary dimension increases, the resonance and anti-resonance frequencies decrease accordingly. However, for a transducer with a center frequency of 40 kHz, using a piezoelectric vibrator in the length mode would result in an excessively large primary dimension. This not only complicates the polarization process of the piezoelectric vibrator but also hinders the miniaturization of the transducer design. To address these challenges, this paper proposes a bending vibration mode based on bimorph coupling, effectively resolving the aforementioned design difficulties.

**Figure 3:**
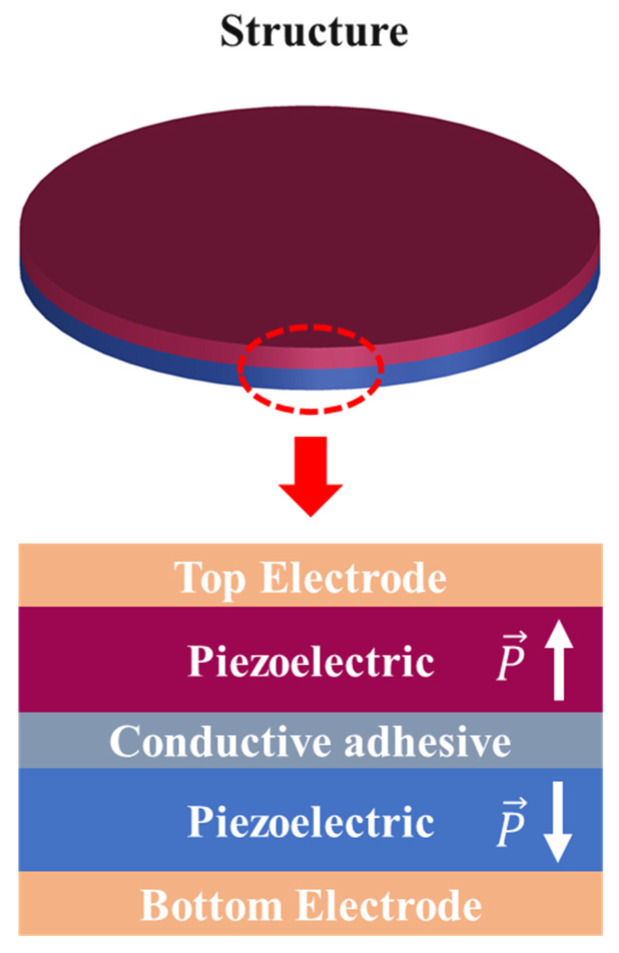
Basic structure of piezoelectric bimorph.

**Figure 4:**
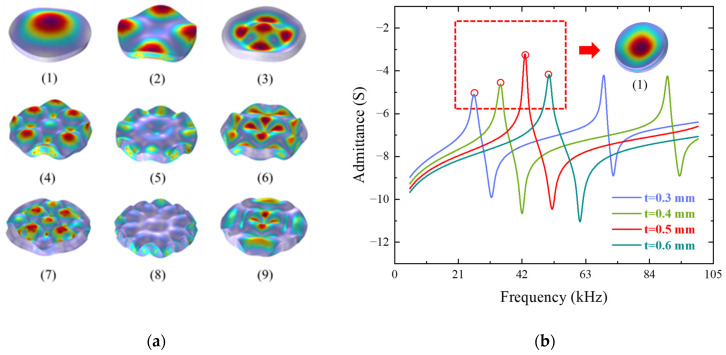
(**a**) First nine bending vibration modes of the piezoelectric bimorph under side free boundary conditions; (**b**) simulation results of impedance characteristic curves of piezoelectric bimorphs of different thicknesses.

The piezoelectric bimorph is composed of two thin piezoelectric plates of identical size, as shown in Figure 3. These two plates are polarized in opposite directions and bonded together using conductive adhesive. When the bimorph vibrates under free boundary conditions, it exhibits multiple vibration modes. Figure 4 illustrates the first nine bending vibration modes of the bimorph. These bending vibration modes can be classified into low-order modes and high-order modes based on their characteristic frequencies. Low-order modes (such as the three modes shown in Figure 4((1)–(3))) typically exhibit simple vibration patterns, with fewer nodes and higher symmetry (e.g., axial symmetry or biaxial symmetry). These modes correspond to lower characteristic frequencies because the simple vibration patterns result in weaker system stiffness and inertial effects. As the mode order increases, the vibration modes become more complex, the number of nodes increases significantly, and the symmetry gradually diminishes, leading to multi-axis symmetry or anti-symmetric modes. Higher-order modes (such as the three modes shown in Figure 4((7)–(9))) feature denser vibration regions, complex and uneven node distributions, and further reduced symmetry. Exciting these complex vibration patterns requires higher characteristic frequencies. Overall, as the mode order increases, the characteristic frequency rises, the vibration pattern becomes more complicated, symmetry decreases, and the number of nodes significantly increases. For receiving transducers, it is essential to convert the received acoustic energy into the vibration modes of the piezoelectric vibrator with the highest efficiency. Evidently, low-order modes, with fewer nodes, stronger symmetry, and easier excitation, are more suitable for this purpose. Therefore, the first vibration mode of the bimorph is selected as the design target for this transducer.

For a specific piezoelectric material, once the working mode and characteristic frequency of the bimorph are determined, its dimensional parameters can be approximately defined. Taking PMN-PT material as an example, when the diameter of the bimorph is set to 10 mm, the admittance curves of bimorphs with different thicknesses can be determined, as shown in Figure 4b. For the same vibration mode, the bimorph’s characteristic frequency shifts toward higher frequencies as the thickness increases. This behavior is the exact opposite of the design principle for vibrators operating in the length mode. In the bending vibration mode of the bimorph, the lower the working frequency of the vibrator, the smaller its size. This further confirms that the proposed design is advantageous for achieving the miniaturization of low-frequency ultrasonic transducers.

### 3.3. Optimal Design of Piezoelectric Bimorph

When operating in the bending vibration mode, the piezoelectric bimorph can generate concentrated vibration responses in specific directions. This design allows precise tuning of the transducer’s anti-resonance frequency by adjusting the diameter-to-thickness ratio of the bimorph, thereby optimizing its sensitivity to the target frequency. The geometric characteristics of the piezoelectric bimorph not only enhance the transducer’s sensitivity to weak signals but also effectively mitigate interference from equipment and environmental noise, especially in complex detection environments. Therefore, an in-depth study of the dimensional parameters of the piezoelectric bimorph is essential to further optimize the transducer’s performance and adaptability.

**Figure 5:**
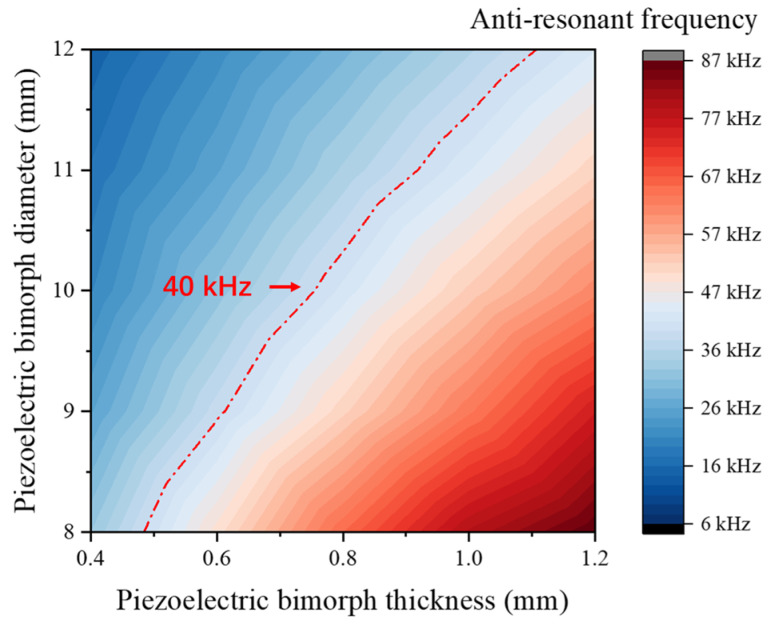
Effect of piezoelectric bimorph thickness on anti-resonance frequency.

**Figure 6:**
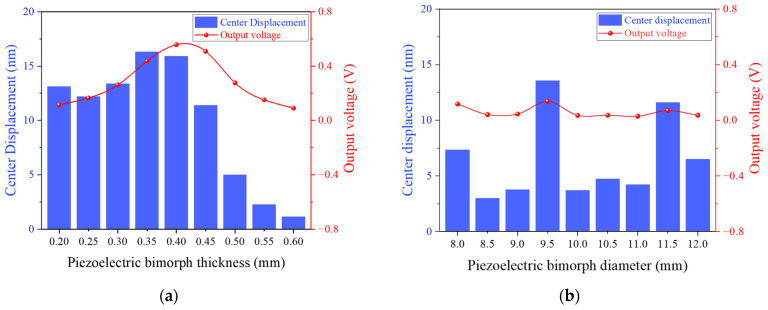
(**a**) Piezoelectric bimorph output maximum displacement and output voltage as a function of thickness; (**b**) piezoelectric bimorph output maximum displacement and output voltage as a function of diameter.

PMN-PT was selected as the piezoelectric material, and the relationship between the dimensions of the bimorph and its anti-resonance frequency was obtained using finite element simulation through parametric scanning of the bimorph’s diameter and thickness, as shown in Figure 5. It is evident that the anti-resonance frequency increases with the thickness of the bimorph and decreases with its diameter. For a specified working frequency, multiple parameter combinations are typically available for selection. To further investigate the influence of thickness on the output response of the piezoelectric bimorph, the diameter was fixed at 10 mm, and a parametric scanning simulation was performed under coupled solid mechanics and electrostatic field conditions. A pressure of 1 Pa was applied to a circular region with a diameter of 1 mm at the center of the bimorph. The simulation results are shown in Figure 6a. Initially, as the thickness increases, the piezoelectric domains contributing to the output response within the bimorph increase, resulting in a larger maximum output displacement and a corresponding rise in output voltage. However, when the thickness exceeds a certain threshold, the mechanical impedance of the bimorph increases with further thickening, leading to a reduction in maximum output displacement and a corresponding decrease in output voltage.

Subsequently, the output responses for different diameters were examined with the thickness fixed at 0.8 mm. The simulation results are shown in Figure 6b. The simulation results show that the output of the bimorph changes slightly when the diameter varies. This indicates that the diameter has a relatively minor impact on the output response of the bimorph, while thickness remains the dominant influencing factor. Therefore, the piezoelectric bimorph was determined to have a diameter of 10 mm and a thickness of 0.8 mm to enhance the transducer’s response sensitivity and voltage output.

### 3.4. Optimal Design of the Horn and Supporting Tube

**Figure 7:**
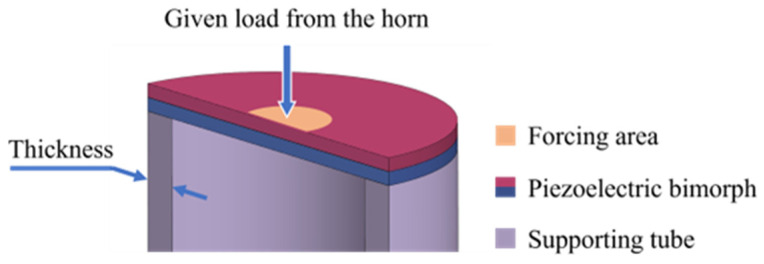
Simulation settings for optimization of the horn and supporting tube.

The primary function of the horn is to concentrate ultrasonic energy from the air and focus it on the center of the piezoelectric bimorph, thereby better exciting the bending vibration mode of the bimorph. The supporting tube serves to provide structural support for the piezoelectric bimorph and works in conjunction with the horn to further excite the bending vibration mode. As shown in Figure 7, the horn transfers ultrasonic signals to a specified load applied to the upper surface of the piezoelectric material, and its forcing area primarily affects the operating state and output performance of the bimorph. Meanwhile, the supporting tube is in contact with the lower surface of the bimorph and provides a supporting force. Due to the annular support surface it offers, the wall thickness of the tube also influences the operating state of the bimorph. Therefore, in the simulation, we investigated the output characteristics of the bimorph under different forcing areas and tube wall thicknesses for a given load, and based on this, we optimized the dimensional parameters of the two components.

**Figure 8:**
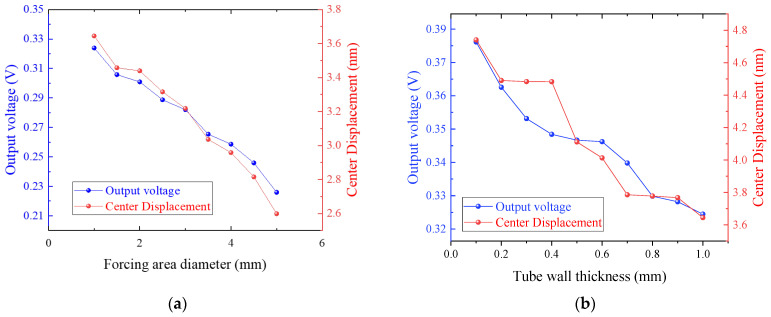
(**a**) Piezoelectric bimorph output maximum displacement and output voltage as a function of horn forcing area diameter; (**b**) piezoelectric bimorph output maximum displacement and output voltage as a function of tube wall thickness.

During simulation, a fixed 1 N total load at a frequency of 40 kHz was applied to the forcing area of the horn to simulate input from acoustic leak signals. The tube wall thickness was set to 1 mm, and the diameter of the forcing area was varied between 1 mm and 5 mm. The piezoelectric bimorph generates a sinusoidal signal during this process, and the amplitude of the sinusoidal signal is defined as the output voltage of the piezoelectric bimorph. The results, shown in Figure 8a, indicate that under the same applied force, as the radius of the forcing area decreases, the output of the piezoelectric bimorph increases, and the central displacement becomes larger. This demonstrates that the more concentrated the applied force, the more effectively the bending vibration mode of the piezoelectric bimorph is excited. Subsequently, an annular fixed constraint was applied to the lower surface of the bimorph to simulate the constraint introduced by the supporting tube. The scanning range for the tube diameter was set between 0.1 mm and 1 mm, while the forcing area diameter was set to 1 mm, with the results shown in Figure 8b. As the tube wall thickness decreases, the output of the piezoelectric bimorph increases, and the central displacement becomes larger, resulting in a more effective excitation of the bending vibration mode.

Although the simulation results indicate that smaller force application areas and thinner tube walls lead to better output performance, practical manufacturing constraints must still be considered during component fabrication and transducer assembly. In the prototype transducer we fabricated, the horn’s bottom diameter was set to 1 mm, and the supporting tube’s wall thickness was set to 0.5 mm.

### 3.5. Transducer Testing Results

**Figure 9:**
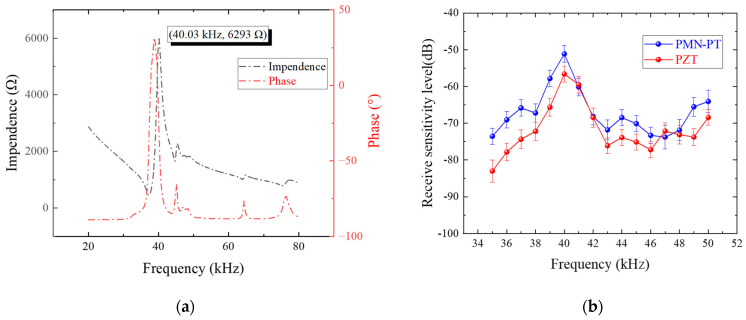
(**a**) Ultrasonic transducer impedance characteristic test; (**b**) receive sensitivity level test results.

The impedance characteristics of the ultrasonic transducer were evaluated, and the results are shown in Figure 9a. The resonance frequency of the transducer was measured to be 37.63 kHz, while the anti-resonance frequency was 40.03 kHz. The measurement results indicate that the fabricated ultrasonic transducer successfully excites a well-defined bimorph bending vibration mode within the frequency range of 36 to 40 kHz. This ensures the stability and sensitivity of the transducer in gas leakage signal detection applications.

The performance of two transducers using PMN-PT and PZT as piezoelectric materials is compared. The standard deviation of the data is derived from ten repeated experiments, and the frequency-dependent sensitivity test results are shown in Figure 9b. Both PMN-PT and PZT transducers show their peak sensitivities at 40 kHz. This peak frequency, shown in Figure 9b, along with the anti-frequency of the transducer in Figure 9a, achieved the target center frequency of 40 kHz, as specified in the design and simulation process demonstrated in Figure 5. Table 2 compares the performance of the self-developed ultrasonic transducer with existing commercial ultrasonic transducers. Evidently, both of our two ultrasonic transducers achieved a higher receiving sensitivity level compared to commercial ones, verifying the superiority of the optimized bending vibration mode used in this study. Furthermore, the PMN-PT transducer outperforms the PZT one with a 5.5 dB higher receive sensitivity level of −51.12 dB. This demonstrates that using PMN-PT as the piezoelectric material can effectively enhance the sensitivity of the transducer designed in this study.

Overall, the transducer fabricated using PMN-PT achieved the design target of a 40 kHz center frequency and effectively improved sensitivity, making it well-suited for gas leakage detection applications. All four transducers exhibit approximately the same bandwidth, with a −6 dB bandwidth around 4 kHz. Transducers using PMN-PT have poorer temperature stability compared to those using PZT, but they can still meet the requirements of gas leak detection applications.

## 4. Discussion

The results of this study highlight the successful design and fabrication of ultrasonic transducers optimized for gas leakage detection applications. The impedance measurements revealed that the fabricated transducer operates within a well-defined bimorph bending vibration mode, achieving stable and sensitive performance within the frequency range of 36–40 kHz. The resonance and anti-resonance frequencies, measured at 37.63 kHz and 40.03 kHz, respectively, confirm the transducer’s ability to achieve the target center frequency of 40 kHz, as specified during the design and simulation process. This frequency alignment is critical for ensuring optimal sensitivity in detecting gas leakage signals.

The comparative analysis between PMN-PT and PZT transducers provides further insights into the advantages of using advanced piezoelectric materials. Both transducers demonstrated peak sensitivity at 40 kHz, consistent with the design target. However, the PMN-PT transducer exhibited a significantly higher receiving sensitivity level (−51.12 dB) compared to the PZT transducer (−56.6 dB), representing a 5.5 dB improvement. This superior performance is attributed to the high piezoelectric properties of PMN-PT and the optimized bimorph bending vibration mode, which enhances the transducer’s ability to detect weak ultrasonic signals. Additionally, the comparison with commercial ultrasonic transducers further validates the effectiveness of the proposed design, as both PMN-PT and PZT transducers outperformed existing products in terms of receiving sensitivity.

From the perspective of previous studies, the use of PMN-PT as a piezoelectric material has been recognized for its high sensitivity and electromechanical coupling coefficient, making it particularly suitable for applications requiring precise signal detection. This study builds upon these findings by integrating PMN-PT into an optimized bimorph bending vibration mode structure, demonstrating its practical advantages in gas pipeline leak detection. Furthermore, the design improvements, including the integration of a horn and supporting tube, effectively enhance the excitation of the bending vibration mode, aligning with previous research on structural optimization for ultrasonic transducers.

The implications of these findings are significant for industrial applications, particularly in ensuring the safe operation of gas pipelines. Gas leakage detection is a critical concern in industrial transportation systems, where timely and accurate detection can prevent environmental hazards and economic losses. The high sensitivity and stable performance of the PMN-PT transducer make it a promising solution for addressing these challenges, enabling more reliable and precise detection of gas leaks.

Future research directions could focus on further enhancing the bandwidth and sensitivity of the transducer to accommodate a wider range of detection scenarios. Additionally, exploring the application of this transducer design in other fields, such as liquid leak detection or structural health monitoring, could expand its utility. Investigating the long-term durability and performance of PMN-PT transducers under varying environmental conditions would also be valuable for ensuring their reliability in real-world applications. Overall, this study lays the foundation for developing advanced ultrasonic transducers with high precision and sensitivity, contributing to safer and more efficient industrial operations.

## 5. Conclusions

Gas pipelines are an essential means of transportation in industrial production, and leak detection is critical for their safe operation. To address this need, this paper proposes a high-sensitivity ultrasonic transducer design method based on a piezoelectric single-crystal material and the bending vibration mode. First, finite element simulations were used to analyze the effects of bimorph dimensions on the bending vibration mode, and the structural parameters of the bimorph were optimized. Then, by integrating a horn and a supporting tube, the bending vibration mode of the bimorph was further excited. The dimensions of both the horn and the supporting tube were further optimized to enhance the transducer’s performance. Through these design improvements, ultrasonic transducers using PMN-PT and PZT were successfully developed and tested. The results show that the center frequency of those self-developed ultrasonic transducers successfully achieves 40 kHz, and their peak receiving sensitivity outperforms similar products. The PZT transducer exhibits a high receive sensitivity level of −56.6 dB, while the PMN-PT one shows a higher sensitivity of −51.1 dB, which benefits from the high-performance piezoelectric material and the bending vibration mode. The transducer thus demonstrates its potential for high-precision gas leak detection in gas pipelines.

## Figures and Tables

**Table 1:** The parameters for the piezoelectric bimorph in COMSOL.

**PMN-PT ((1 − x)Pb(Mg_1/3_Nb_2/3_O_3_ − xPbTiO_3_, x = 0.29)**	
Density (kg/m^3^)	8050
Relative permittivity $\left[\begin{array}{ccc} \varepsilon_{11}^{S}/\varepsilon_{0} & & \\ & \varepsilon_{22}^{S}/\varepsilon_{0} & \\ & & \varepsilon_{33}^{S}/\varepsilon_{0} \end{array}\right]$	$\left[\begin{array}{ccc} 1370 & & \\ & 1370 & \\ & & 977 \end{array}\right]$
Elastic Matrix ($10^{10}\;N/m^{2}$) $\left[\begin{array}{cccccc} c_{11}^{E} & c_{12}^{E} & c_{13}^{E} & c_{14}^{E} & c_{15}^{E} & c_{16}^{E}\\ & c_{22}^{E} & c_{23}^{E} & c_{24}^{E} & c_{25}^{E} & c_{26}^{E}\\ & & c_{33}^{E} & c_{34}^{E} & c_{35}^{E} & c_{36}^{E}\\ & & & c_{44}^{E} & c_{45}^{E} & c_{46}^{E}\\ & & & & c_{55}^{E} & c_{56}^{E}\\ & & & & & c_{66}^{E} \end{array}\right]$	$\left[\begin{array}{cccccccc} 15.74 & 14.21 & 11.42 & & 0 & 0 & 0 & \\ & 15.74 & 11.42 & & 0 & 0 & 0 & \\ & & 10.45 & & 0 & 0 & 0 & \\ & & & 7.35 & & 0 & & 0\\ & & & & & 7.35 & & 0\\ & & & & & & & 5.85 \end{array}\right]$
Coupling Matrix ($C/m^{2}$) $\left[\begin{array}{cccccc} e_{11} & e_{12} & e_{13} & e_{14} & e_{15} & e_{16}\\ e_{21} & e_{22} & e_{23} & e_{24} & e_{25} & e_{26}\\ e_{31} & e_{32} & e_{33} & e_{34} & e_{35} & e_{36} \end{array}\right]$	$\left[\begin{array}{cccccc} 0 & 0 & 0 & 0 & 4.54 & 0\\ 0 & 0 & 0 & 4.54 & 0 & 0\\ -3.03 & -3.03 & 24.03 & 0 & 0 & 0 \end{array}\right]$

**Table 2:** Comparison of performance parameters of different air-coupled ultrasonic transducers.

Transducer Type	Material	Center Frequency (kHz)	−6 dB Bandwidth (kHz)	Receive Sensitivity Level (ref. V/μbar)	Working Temperature (°C)
This work	PMN-PT	40	4	−51.1 dB	40~80
This work	PZT	40	4	−56.6 dB	−25~100
SAIPUS N4100	PZT	40	4	−63 dB	−25~100
PXR04A	PZT	40	4	−64 dB	−25~100

## Data Availability

Data is contained within the article.
